# Exploring the Link between Inflammatory Biomarkers and Adipometrics in Healthy Young Adults Aged 20–35 Years

**DOI:** 10.3390/nu16020257

**Published:** 2024-01-15

**Authors:** Irina Bianca Kosovski, Vladimir Bacârea, Dana Ghiga, Cristina Nicoleta Ciurea, Dragos Constantin Cucoranu, Adina Hutanu, Anca Bacârea

**Affiliations:** 1 Department of Pathophysiology, George Emil Palade University of Medicine, Pharmacy, Science and Technology of Târgu Mureș, 540139 Târgu Mureș, Romania; bianca.kosovski@umfst.ro (I.B.K.); anca.bacarea@umfst.ro (A.B.); 2 Doctoral School, George Emil Palade University of Medicine, Pharmacy, Science and Technology of Târgu Mureș, 540139 Târgu Mureș, Romania; 3 Department of Research Methodology, George Emil Palade University of Medicine, Pharmacy, Science and Technology of Târgu Mureș, 540139 Târgu Mureș, Romania; dana.ghiga@umfst.ro; 4 Department of Microbiology, George Emil Palade University of Medicine, Pharmacy, Science and Technology of Târgu Mureș, 540139 Târgu Mureș, Romania; cristina.ciurea@umfst.ro; 5 Department of Radiology, Mures County Emergency Hospital, 540136 Târgu Mureș, Romania; cucoranud@gmail.com; 6 Center for Advanced Medical and Pharmaceutical Research, George Emil Palade University of Medicine, Pharmacy, Sciences and Technology Târgu Mureș, 540139 Târgu Mureș, Romania; adina.hutanu@umfst.ro

**Keywords:** obesity, inflammation, healthy young adults, cytokines, body composition, anthropometric measurements, hsCRP

## Abstract

Obesity and aging are associated with an inflammatory state, which represents the common background for a wide range of diseases. This study aims to explore the correlation between hsCRP, IL-1β, IL-6, TNF-α, IFN-γ, and white blood cell count (WBC) and adipometrics (arm, waist, and hip circumferences: AC, WC, HC; total body fat mass: TBFM, visceral fat level: VFL, body mass index: BMI; waist/hip ratio: WHR; waist/height ratio: WHtR) in young and healthy adults aged 20–35 years old. The subjects were divided by BMI into the overweight/obesity (OW/OB) group and normal weight (NW) group, and by hsCRP level into Group 1 (<1 mg/L), Group 2 (≥1–2.99 mg/L), and Group 3 (≥3 mg/L). The concentration of all inflammatory biomarkers was significantly higher in the OW/OB group compared to the NW group, with the exception of IL-1β. Significant positive correlations were found between hsCRP, TNF-α, WBC, and all adipometrics; between IL-6 and WHR, WHtR, BMI, TBFM, and VFL; and between IFN-γ and HC, BMI, and TBFM. IL-1β correlates positively with WHR and VFL. In Groups 1–3, all the differences between the adipometrics showed significant differences. Subclinical inflammation persists in association with being overweight and obese in healthy young adults aged 20–35 years old.

## 1. Introduction

Obesity has become a global public health challenge, transcending geographical boundaries. According to the 2022 report from the World Health Organization (WHO), obesity is no longer a problem in developed countries and no nation is immune to this epidemic [1]. Obesity is correlated with a sedentary lifestyle associated with fast-food intake, a classic behavior link of our era [2]. Moreover, after COVID-19 restrictions were in place, the prevalence of obesity increased even more [3,4,5].

In obesity, hypertrophied adipocytes within the adipose tissue (AT) induce hypoxia due to inadequate vascularization, resulting in cell death primarily through necrosis rather than apoptosis. This process initiates local events within AT, including leptin secretion, cellular infiltration (neutrophils, T cells, macrophages), and the release of inflammatory cytokines [6,7]. Furthermore, >90% of all macrophages situated in the white AT of obese mice and humans are located near adipocytes undergoing necrotic-like changes [6]. There is also a positive correlation between adipocyte size and rate of death. Macrophages scavenge the adipocyte debris with secondary formation of macrophage syncytia, which is associated with an increased TNF-α gene expression at the level of adipocytes [6]. These modifications mark the initial phase of obesity-related inflammation, while the systemic extension represents the onset of chronic low-grade inflammation (LGI).

LGI represents the background of numerous noncommunicable diseases (NCDs), impacting multiple body systems [8]. In Europe, 60% of adults are overweight or obese, representing the fourth most common risk factor for NCDs [5]. Traditionally, inflammation is an early, nonspecific protective mechanism against body injuries that ensures the restoration of the body’s homeostasis. Nevertheless, if inflammation exceeds its protective role, the patient is predisposed to a large spectrum of acute and chronic pathologies, making inflammation a double-edged sword [9]. Compared with classic inflammation, during LGI, the serum levels of pro-inflammatory cytokines are slightly and constantly elevated, remaining asymptomatic even if the same signaling pathways are activated [8,10]. The impact on the risk of developing NCDs and metabolic disorders varies according to the anatomic distribution of fat deposition. Compared with subcutaneous fat, visceral fat is strongly associated with arterial inflammation and insulin residence based on the specific inflammatory pattern [11,12,13].

No cut-off values for cytokines are described in the literature to clearly define LGI [14]. However, the aging process is linked with immunosenescence and inflammaging, both of which contribute to significant alterations in the immune system response and increased levels of circulating inflammatory markers [8,15,16]. To characterize LGI primarily associated with AT, a young, healthy, and hormonally and metabolically stable population is required. Therefore, this study aims to evaluate the correlation between hsCRP, IL-1β, IL-6, TNF-α, IFN-γ, and white blood cell count (WBC) as pro-inflammatory cytokines, and adipometrics (arm, waist, and hip circumferences, total body fat mass, visceral fat level, BMI, waist-to-hip ratio, and waist-to-height ratio) in healthy young adults aged 20–35 years old.

## 2. Materials and Methods

### 2.1. Study Design and Population

This cross-sectional study was approved by the Ethics Committee of the County Clinical Emergency Hospital of Târgu Mureș (Decision no. Ad.29270/08.12.2020) and informed written consent was obtained from each subject. The study adheres to the guidelines outlined in the Declaration of Helsinki.

The inclusion criteria comprised individuals aged between 20 and 35 years old who were clinically healthy. We define clinically healthy as individuals without acute or chronic inflammatory/infectious diseases, cancers, or pregnant or postpartum status, or the use of chronic medication such as anti-inflammatory drugs or other types of agents. A triage questionnaire was applied, collecting information on individuals’ personal and family history, as well as medication details.

### 2.2. Laboratory Methods

#### 2.2.1. Blood Sample Collection

Venous blood samples were collected in the morning following an 8–10 h fast. BD Vacutainers^®^ (Becton, Dickinson and Company, Franklin Lakes, NJ, USA) with a clot activator and gel serum separator, as well as K3 EDTA, were utilized. After 30 min of blood collection, serum isolation and hematology tests were performed. Due to variations in the processing times of the inflammatory markers, each serum sample was aliquoted into three 1.5 mL conical Eppendorf^®^ tubes (Eppendorf, Hamburg, Germany) and subsequently stored at −80 degrees Celsius.

#### 2.2.2. Multiplex Bead-Based Immunoassay

Serum concentrations of IL-1β, IL-6, and TNF-α were simultaneously measured using Luminex^®^ xMAP^®^ technology (a multiplex bead-based immunoassay) with the MILIPLEX^®^ Human Adipokine Magnetic Bead Panel 2 kit, HADK2MAG-61K (Merck KGaA, Darmstadt, Germany; MilliporeSigma, Seattle, WA, USA). The FLEXMAP 3D^®^ analyzer (Luminex Corporation, Austin, TX, USA, a DiaSorin Company) and xPONENT^®^ software version 4.3 were used to quantify and analyze the obtained data. The results were expressed as pg/mL.

#### 2.2.3. The Enzyme-Linked Immunosorbent Assay

IFN-γ was measured using the enzyme-linked immunosorbent assay (ELISA) with the Quantikine^®^ ELISA Human IFN-γ kit (R&D System, Inc., Minneapolis, MN, USA) following the manufacturer’s instructions. Readings were performed using the Elisa Dynex DSX analyzer (DYNEX^®^ TECHNOLOGIES, Chantilly, VA, USA). Concentrations were calculated based on a 7-point standard curve provided with the kit, and the results were expressed in pg/mL.

#### 2.2.4. Turbidimetric Assay

The hsCRP was quantified using the turbidimetric assay with the COBAS INTEGRA CRP HS kit (cat. No. 04628918190, ROCHE) read on a COBAS INTEGRA 400 plus analyzer (ROCHE, F. Hoffmann–La Roche, Ltd., Basel, Switzerland). The results were expressed as mg/L.

#### 2.2.5. White Blood Cell Count

The white blood cell count (WBC) was assessed with a Sysmex^®^ analyzer (Sysmex Corporation, Kobe, Japan) based on fluorescent flow cytometry with a semiconductor laser and hydrodynamic focusing. The results were expressed as cells ×10^3^/µL.

### 2.3. Anthropometric Measurements

Anthropometric direct measurements included arm, waist, and hip circumferences (AC, WC, HC—cm) and height (cm). AC was measured midway between the acromion and olecranon on the shoulder blade and on the ulna of the arm. WC was measured at the midpoint between the upper iliac spine and the last ipsilateral rib, determined along the midaxillary line. HC was assessed at the level of the femoral tuberosities. The waist-to-hip ratio (WHR) and waist-to-height ratio (WHtR) were calculated.

TANITA BC-1000 Inner Scan^®^ scale (TANITA Corporation, Tokyo, Japan), utilizing bioelectrical impedance analysis, was used to assess total body fat mass (kg) (TBFM), visceral fat level (VFL), weight (kg), and BMI. All subjects were dressed in light clothing but without shoes and socks to optimize contact with the electrodes. BMI was calculated according to the WHO formula: BMI = weight/height^2^ (kg/m^2^) [17]. Participants were divided into groups of being a normal weight (NW) (18.5–24.9), overweight (OW) (25–29.9), and obese (OB) (≥30.0), according to their BMI. Adipometrics were defined: AC, WC, HC, WHR, WHtR, BMI, TBFM, and VFL.

### 2.4. Statistical Methods

The statistical analysis included descriptive statistics (frequency, percentage, mean, median, standard deviation—SD) and elements of inferential statistics. The Shapiro–Wilk test was applied to determine the distribution of the analyzed data series. The Mann–Whitney U test, a non-parametric test for comparison of medians, was applied. Post hoc tests were applied for each statistical analyses as follows: for variables with a Gaussian distribution where the ANOVA test was applied, we used the Bonferroni test, and for those with a non-Gaussian distribution (Kruskal–Wallis test), the Dunn test was used. The Spearman test was used to evaluate the correlation (measures the strength of association) between quantitative variables, and the Chi-square test and the Fisher test were used to determine the association between the qualitative variables. The significance threshold chosen for the *p*-value was 0.05. Statistical analyses were performed using the trial version of the GraphPad Prism trial version.

## 3. Results

### 3.1. Cohort Classification and Analyses Based on BMI

A total of 128 out of 167 participants met the eligibility criteria for the study. General characteristics of the cohort classified based on BMI are presented in Table 1. They were divided into an overweight/obese (OW/OB, 91, 71.09%) group and a normal-weight (NW, 37, 28.91%) group, according to BMI (≥25.0, 18.5–24.9, respectively). The age distribution of the groups was similar, with a median age of 28.0 years for the OW/OB group and 27.0 years for the NW group. The OW/OB group included 54 males (58.24%) and 38 females (41.76%), with the preponderance originating from urban areas (86.67%). The NW group consisted of 7 males (18.92%) and 30 females (81.08%), with 83.78% of them residing in urban areas.

The differences in adipometrics and inflammatory cytokine concentration according to BMI are presented in Table 2. BMI and TBFS, as measures of the total body adipose tissue distribution, were significantly higher in the OW/OB group compared to the NW (*p* < 0.0001) group, at 30.89 vs. 21.35 and 31.03 vs. 13.67, respectively. Furthermore, all indices used to identify and quantify regional adipose tissue deposition, such as VFL, AC, WC, and HC, were also significantly higher in the OW/OB (*p* < 0.0001) group. Additionally, both WHR and WHtR presented significantly higher mean values in the OW/OB group compared to the NW (*p* < 0.0001) group, at 0.86 vs. 0.75 and 0.56 vs. 0.43, respectively.

Overall, the concentrations of all inflammatory cytokines were higher in the OW/OB group compared to the NW group. A significantly higher concentration of IL-6 and hsCRP was observed in the OW/OB group compared to the NW group, at 19.49 vs, 13.42 (*p* = 0.0201) and 2.46 vs. 0.53 (*p* < 0.0001). Furthermore, TNF-α and IFN-γ were significantly higher in the OW/OB group, 5.63 vs. 3.44 and 21.35 vs. 19.63 (*p* < 0.0001, *p* = 0.0216), respectively. However, WBC followed a similar trajectory, demonstrating a significant elevation in the OW/OB group, 6.91 vs. 6.07 (*p* = 0.0016). However, IL-1β, despite having a higher concentration in the OW/OB group, was the only marker that did not present a significant difference between the groups (*p* = 0.8746).

We also evaluated whether inflammatory cytokines and adipometrics were related, and the correlations are presented in Table 3. We found that the higher the adipometrics, the higher the median concentration of the IC in the entire study group, except for IL-1β. Also, hsCRP, TNF-α, and WBC showed significant positive correlations with all adipometrics (AC, WC, HC, WHR, WHtR, BMI, TBFM, VFL). Additionally, IL-6 presented a significant positive correlation with WHR, WHtR, BMI, TBFM, and VFL (*p* < 0.05, *p* < 0.005, *p* < 0.05, *p* < 0.05), while IFN-γ showed a significant positive correlation with HC, BMI, and TBFM (*p* < 0.05). Although IL-1β did not present any significant correlation, it exhibited two trends: a weak positive correlation with WHR and VFL, and a negative correlation with AC, WC, HC, WHtR, BMI, and TBFM.

Additionally, when we analyzed the entire cohort, we found a positive correlation between IL-6 and hsCRP (r = 0.0380; *p* = 0.6700), and TNF-α and IFN-γ (r = 0.1712; *p* = 0.0533).

### 3.2. Cohort Classification and Analyses Based on hsCRP

To better investigate the subclinical inflammatory syndrome, a second classification of the cohort was made based on hsCRP concentration, dividing individuals into three groups: Group 1, <1 mg/L (71, 55.46%); Group 2, ≥1–2.99 mg/L (37, 28.9%); and Group 3, ≥3 mg/L (20, 15.62%). The comparisons between the adipometrics and the groups are detailed in Table 4. All the medians of the adipometrics showed significant differences, with a constant increase from Group 1 to Group 3 (*p* < 0.05). Furthermore, when comparing each pair of groups (1 vs. 2; 1 vs. 3; 2 vs. 3) we observed the same significant differences (*p* < 0.05) in all cases, with a single exception, and post hoc tests confirmed the observed differences. The exception is represented by AC Group 2 vs. AC Group 3, where the Dunn test indicates that the difference is nonsignificant, as can be seen in Table 4.

## 4. Discussion

The participants included in the study were aged between 20 and 35 years old, as an attempt to analyze obesity-associated inflammation as accurately as possible. This selection aims to exclude the potential effects of immunosenescence and inflammaging which could otherwise impact the baseline levels of inflammatory markers. On the other hand, chronic treatment, which is commonly prescribed as an individual advances in age, can also influence the baseline secretion. For a meaningful comparison of our findings, it is recommended to take into consideration studies with a comparable age group and inclusion criteria, whenever possible.

CRP is an acute-phase protein whose secretion is induced by IL-6, primarily in hepatocytes [18]. To identify LGI, hsCRP is the most commonly used marker due to the availability of standardized methods and its stability [19]. In our study, although hsCRP levels within the reference range (<5 mg/L) were noted in both groups, a significant difference persisted between them (*p* < 0.0001). Nevertheless, an elevated hsCRP level within the reference range among apparently healthy subjects was linked to an increased risk of developing cardiovascular disease (CVD), diabetes mellitus, and non-alcoholic fatty liver disease, with a cut-off value of 3 mg/L [19,20,21]. We also obtained significant differences between the groups divided by hsCRP levels, with Group 3 presenting the highest median adipometric values. Since 1998, a strong correlation has been described between the size of fatty streaks in the aorta (60% prevalence) and coronary arteries (69% prevalence) with BMI in young adults aged 26–39 years [22].

IL-6 is a pleiotropic cytokine present in both immune responses (by stimulating B lymphocytes) and nonimmune processes such as acute or chronic inflammation, autoimmune disorders, and even cancer [23,24]. However, our results showed that the median concentration of IL-6 was significantly higher in the OW/OB group compared to the NW group (3.36 vs. 0.71, *p* = 0.0201). Interestingly, there did not appear to be the same significant correlation between IL-6 and hsCRP and the adipometrics; this was as expected, considering their secretion mechanism; hsCRP presented a stronger positive correlation with all adipometrics when compared with IL-6.

Previous studies have also confirmed our results. A positive and significant correlation was found between IL-6 and BMI in Egyptian adults with grade III obesity, using approximately the same inclusion criteria as ours [25]. CVD risk has been associated with a lower BMI level for Asian individuals compared to a Western cohort. However, a study including apparently healthy Korean adults reported significant correlations between CRP, TNF-α, and IL-6 with weight, BMI, WC, HC, and WHR. While TNF-α presented a positive correlation with these anthropometric markers, the correlation was not significant. In the obese group, CRP was significantly associated with BMI, whereas IL-6 was significantly related to visceral adiposity [26].

IL-1β is a proinflammatory marker that is firstly secreted in the inactive form as pro-IL-1β, which is cleaved by caspase 1 into the active IL-1β. The disease spectrum induced by IL-1β is broad and includes microbial infection, autoimmune disorders (Crohn’s disease, rheumatoid arthritis, systemic lupus erythematosus, etc.), osteoarthritis, neurodegenerative diseases, insulin resistance, and LGI [27,28,29,30]. Furthermore, in mice models, IL-1β signaling increases hepatic lipogenesis and steatosis by upregulating fatty acid synthase, thus highlighting the link between non-alcoholic fatty liver disease and obesity [31].

IL-1β shows a significant positive correlation with HbA1c, being an inflammatory marker associated with the malfunction of pancreatic islets under in vitro conditions [32]. Therefore, studies have shown that inhibition of IL-1β increases insulin sensitivity, improves renal function, and reduces cardiovascular complications in patients with chronic kidney disease [33,34].

In our study, IL-1β concentration was higher in the OW/OB group, being the only marker showing no significant difference. Borges MD et al. found the highest levels of IL-1β also occurred in the obese group, compared with the other BMI categories [35]. Among obese adolescent girls aged 13–18 years old, a significantly higher concentration of IL-1β was found in the group with central obesity (WHR > 0.8), along with a positive correlation between WHR and IL-1β [36].

In our study, IL-1β showed a weak and insignificant negative correlation with all indices representing indicators of obesity, except the WHR and VFL. IL-1β is expressed more in the visceral adipose tissue (VAT) compared to the subcutaneous adipose tissue (SAT) based on the increased activity of the NLR family pyrin domain containing 3 (NLRP3) inflammasome which leads to increased activation of caspase 1 activity in VAT [37,38,39]. In addition, the expression of the IL-1β mRNA level is increased in VAT compared to SAT [40]. As WHR and VFL are both indices that better express the VAT, the positive correlation can be explained.

The physiological role of IFN-γ is to enhance the immune system activity against microbial infections and neoplastic cells, conferring protection in infectious diseases and cancer [41,42]. However, its overproduction results in sustained inflammation, tissue damage, and eventual necrosis, thereby contributing to the pathogenesis of multiple diseases such as autoimmune, atopic, or neurodegenerative diseases [43,44,45]. In adipose tissue, T lymphocytes and NK cells are the primary sources of IFN-γ secretion [46]. In a murine model study comparing obese IFN-γ-knockout mice vs. mice with diet-induced obesity, the roles of IFN-γ were demonstrated in the regulation of the AT structure (enlarged adipocyte and leukocytic infiltration), M2 shift of AT macrophage phenotype, cytokine expression (increased TNF-α and decreased IL-10 transcription levels), and reduced insulin sensitivity in mice with diet-induced obesity [47]. On the other hand, an in vitro study of stromovascular cell fractions from obese individuals undergoing bariatric surgery demonstrated the same correlations observed in the murine model. Furthermore, IFN-γ secretion was associated with increased TNF-α expression in macrophages [46]. In our study, we also found a positive correlation between the serum levels of IFN-γ and TNF-α (r = 0.1712; *p* = 0.0533). Additionally, significant positive correlations were found between HbA1c and IFN-γ, as well as TNF-α (r = 0.32, *p* = 0.04; r = 0.30, *p* = 0.05) in an African American young obese female cohort [32]. Using a similar cohort to that used in our study, a recent study reported the same significant increase in the level of IFN-γ in the OW/OB group. They explained that the decreased frequency of regulatory B lymphocytes increases the levels of Th1/Th17 cytokines [48].

TNF-α is primarily known as a pro-inflammatory cytokine, produced mainly by macrophages. In the context of obesity, the presence of macrophage infiltration in AT, can explain the increased level of TNF-α in obese individuals [49]. TNF-α plays a complex role in obesity, being associated with insulin resistance/type 2 diabetes, alteration in adipogenesis, increased lipolysis, and amplification of systemic inflammation by stimulating the production of other cytokines such as MCP-1 and IL-6 [50,51]. In our study we identified a significant increase in TNF-α concentration in the OW/OB group, along with strong correlations between TNF-α and all of the adipometrics (*p* < 0.05). Furthermore, an elevated serum concentration of TNF-α was observed in the obese group compared with the non-obese group (2.69 vs. 1.72 pg/mL) in a study, demonstrating a strong correlation between TNF-α and weight, BMI, waist and hip circumferences, as well as WHR (*p* < 0.05) [26].

Even though WBC is not a classic marker of LGI, we observed a significantly increased level in the OW/OB group compared to the NW group and a significant positive correlation with all adipometrics. In accordance with our results, a study involving a cohort of young healthy women reported a significantly positive relationship between WBC and body fat (%), WC, and CRP, but not with IL-6 [52]. Additionally, other studies reported the same differences and correlations between WBC and BMI in apparently healthy young adults and even in adolescents [53,54]. Furthermore, an elevated WBC within the normal range can predict the incidence of and serve as an independent risk factor for type 2 diabetes, hypertension, and MS among young obese individuals [55,56,57,58]. Even when it was within the reference range, WBC > 7.6 × 10^9^ cells/L was an independent predictor of cardiovascular mortality [59]. On the other hand, the clinical value of WBC in assessing obesity-related inflammation and its side effects is limited, and classic inflammatory cytokines are still more likely to provide useful information [60]. Additionally, WBC also increases with age [54].

Since BMI calculation includes weight and height and may not necessarily correlate with the quantity of adipose tissue, additional indices are used to comprehensively evaluate body fat distribution [61,62]. Compared to subcutaneous adipose tissue (SAT), numerous studies have demonstrated that visceral adipose tissue (VAT) increases the risk of obesity-related diseases as well as overall mortality based on unique inflammatory patterns [12,13,63,64]. Nevertheless, in severe obesity, abdominal SAT appears to play a greater role in the pro-inflammatory environment compared to intra-abdominal VAT, presenting greater gene expression of numerous cytokines [65]. Therefore, a clear conclusion remains: regardless of the AT location; if it is in excess, inflammation is inevitable.

For a comprehensive understanding of the results, they need to be interpreted in the context of the study’s limitations. One limitation is represented by the relatively small size of the cohort and the unique blood sample determination of the inflammatory biomarkers used to assess an individual’s long-term state of inflammation. Moreover, the study design did not establish the temporal relationship between exposure and outcome. This limitation restricts the study’s capacity to identify trends and patterns over time. Another limitation is represented by the imbalanced distribution of sexes within the NW group, which can interfere with the final results. Additionally, as a prospect for future research in this medical context, it is advisable to consider reciprocal measurements of anti-inflammatory cytokines, such as IL-10, IL-11, or IL-13, for an exhaustive study of a subclinical inflammatory syndrome.

## 5. Conclusions

In conclusion, this study contributes valuable insights into the early dynamics of inflammation among young, apparently healthy adults. Based on the significant positive correlation of the adipometrics with cytokine levels, our data indicate that low-grade inflammation is present independently of age and subsequent pathologies, even in the apogee of life. The risk of developing obesity-related pathologies remains consistent, and these findings demonstrate that decreasing the quantity of adipose tissue may represent a valuable preventive measure.

## Figures and Tables

**Table 1:** General characteristics of the cohort classified based on BMI.

		Overweight and Obese (n = 91)	Normal Weight (n = 37)
Age—Mean ± SD (Median)		28.08 ± 4.66 (28.00)	28.03 ± 3.63 (27.00)
Sex	male—n (%)	53 (58.24%)	7 (18.92%)
	female—n (%)	38 (41.76%)	30 (81.08%)
Provenience	urban—n (%)	78 (86.67%)	31 (83.78%)
	rural—n (%)	12 (13.33%)	6 (16.22%)

**Table 2:** Comparison of adipometrics and concentrations of the inflammatory cytokines between the study and control groups.

	Overweight and Obese (n = 91) Mean ± SD (Median)	Normal Weight (n = 37) Mean ± SD (Median)	*p* *
The adipometrics			
BMI	30.89 ± 5.36 (28.80)	21.35 ± 1.82 (21.20)	<0.0001
Total body fat mass (kg)	31.03 ± 13.38 (28.20)	13.67 ± 3.89 (13.40)	<0.0001
Visceral fat level	8.65 ± 5.07 (7.00)	2.00 ± 1.08 (2.00)	<0.0001
Arm circumference (cm)	32.80 ± 4.00 (32.00)	26.59 ± 3.14 (26.00)	<0.0001
Waist circumference (cm)	98.48 ± 14.19 (97.00)	73.70 ± 9.18 (72.00)	<0.0001
Hip circumference (cm)	113.20 ± 9.90 (111.00)	97.65 ± 4.83 (97.00)	<0.0001
Waist-to-hip ratio	0.86 ± 0.08 (0.87)	0.75 ± 0.07 (0.72)	<0.0001
Waist-to-height ratio	0.56 ± 0.07 (0.55)	0.43 ± 0.04 (0.42)	<0.0001
The inflammatory cytokines			
hsCRP (mg/L)	2.46 ± 3.55 (1.15)	0.53 ± 0.51 (0.38)	<0.0001
IL-1β (pg/mL)	0.89 ± 1.70 (0.42)	0.78 ± 0.93 (0.46)	0.8746
IL-6 (pg/mL)	19.49 ± 42.89 (3.36)	13.42 ± 37.68 (0.71)	0.0201
TNF-α (pg/mL)	5.63 ± 4.30 (4.92)	3.44 ± 1.43 (3.44)	<0.0001
IFN-γ (pg/mL)	21.35 ± 3.40 (21.84)	19.63 ± 2.51 (18.77)	0.0216
WBC (10^3^/µL)	6.91 ± 1.58 (6.57)	6.07 ± 1.32 (5.92)	0.0016

BMI—body mass index; hsCRP—high-sensitivity C-reactive protein; IL—interleukin; TNF-α—tumor necrosis α; IFN-γ—interferon γ; WBC—white blood cell count; SD—standard deviation. * *p*-value obtained with the Mann–Whitney U test.

**Table 3:** Correlations between inflammatory cytokines and adipometrics in all subjects.

	AC	WC	HC	WHR	WHtR	BMI	TBFM	VFL
	r (CI 95%)	r (CI 95%)	r (CI 95%)	r (CI 95%)	r (CI 95%)	r (CI 95%)	r (CI 95%)	r (CI 95%)
hsCRP (mg/L)	0.5238 (0.3808 to 0.6423) ***	0.5389 (0.3986 to 0.6545) ***	0.5332 (0.3920 to 0.6499) ***	0.4361 (0.2793 to 0.5703) ***	0.6005 (0.4726 to 0.7037) ***	0.6419 (0.5233 to 0.7361) ***	0.6369 (0.5171 to 0.7322) ***	0.5970 (0.4683 to 0.7008) ***
IL-1β (pg/mL)	−0.0507 (−0.2273 to 0.1290)	−0.03595 (−0.2132 to 0.1436)	−0.0645 (−0.2404 to 0.1153)	0.0135 (−0.1654 to 0.1917)	−0.0091 (−0.1874 to 0.1697)	−0.0058 (−0.1843 to 0.1729)	−0.0379 (−0.2151 to 0.1416)	0.0387 (−0.1408 to 0.2158)
IL-6 (pg/mL)	0.0924 (−0.0875 to 0.2666)	0.1734 (−0.0053 to 0.3414)	0.1212 (−0.0586 to 0.2934)	0.2026 (0.0248 to 0.3679) *	0.2540 (0.0789 to 0.4138) **	0.2265 (0.0499 to 0.3894) *	0.1979 (0.0200 to 0.3636) *	0.2354 (0.0593 to 0.3973) *
TNF-α (pg/mL)	0.2771 (0.1036 to 0.4342) **	0.3356 (0.1670 to 0.4851) ***	0.2619 (0.0873 to 0.4208) **	0.3089 (0.1379 to 0.4620) **	0.2973 (0.1253 to 0.4519) **	0.2499 (0.0746 to 0.4102) **	0.1839 (0.0054 to 0.3510) *	0.3002 (0.1284 to 0.4544) **
IFN-γ (pg/mL)	0.1695 (−0.0093 to 0.3379)	0.1518 (−0.0275 to 0.3217)	0.2149 (0.0377 to 0.3790) *	0.0774 (−0.1026 to 0.2525)	0.1401 (−0.0395 to 0.3109)	0.1977 (0.0197 to 0.3634) *	0.1814 (0.0028 to 0.3487) *	0.1661 (−0.0128 to 0.3348)
WBC (×10^3^/µL)	0.2405 (0.0646 to 0.4018) *	0.3097 (0.1388 to 0.4627) **	0.4417 (0.2856 to 0.5749) ***	0.1863 (0.0079 to 0.3531) *	0.3327 (0.1639 to 0.4826) ***	0.3799 (0.2159 to 0.5230) ***	0.4058 (0.2450 to 0.5449) ***	0.3187 (0.1485 to 0.4705) **

hsCRP—high-sensitivity C-reactive protein; IL—interleukin; TNF-α—tumor necrosis α; IFN-γ—interferon γ; WBC—white blood cell count; AC—arm circumference; WC—waist circumference; HC—hip circumference; WHR—waist-to-hip ratio; WHtR—waist-to-height ratio; BMI—body mass index; TBFM—total body fat mass; VFL—visceral fat level; CI—confidence interval; r—correlation coefficient. Correlations were calculated with the Spearman test. * *p* < 0.05; ** *p* < 0.005; *** *p* < 0.0001.

**Table 4:** Comparisons between the adipometrics in the hsCRP groups.

	Group 1 (n = 71) Mean ± SD (Median)	Group 2 (n = 37) Mean ± SD (Median)	Group 3 (n = 20) Mean ± SD (Median)	*p*-Value	Post Hoc Test *p* < 0.05
AC	29.08 ± 3.88 (29.00)	32.22 ± 4.17 (32.00)	35.60 ± 4.52 (37.00)	<0.0001 *	Group 2 vs. Group 3 NS ^‡^
WC	84.92 ± 14.36 (86.00)	93.86 ± 14.84 (93.00)	109.40 ± 16.71 (107.50)	<0.0001 *	Yes ^‡^
HC	104.40 ± 8.45 (104.00)	110.60 ± 11.18 (109.00)	120.40 ± 11.02 (119.50)	<0.0001 ⸷	Yes ^§^
WHR	0.80 ± 0.09 (0.81)	0.84 ± 0.10 (0.84)	0.90 ± 0.08 (0.92)	0.0005 *	Yes ^‡^
WHtR	0.49 ± 0.06 (0.49)	0.54 ± 0.07 (0.54)	0.62 ± 0.08 (0.63)	<0.0001 *	Yes ^‡^
BMI	25.12 ± 4.26 (25.30)	29.51 ± 5.16 (28.70)	36.30 ± 6.59 (37.95)	<0.0001 *	Yes ^‡^
TBFM	18.20 ± 7.52 (18.20)	26.30 ± 10.95 (26.30)	45.20 ± 17.53 (45.20)	<0.0001 *	Yes ^‡^
VFL	4.52 ± 3.08 (4.00)	7.62 ± 5.11 (7.00)	12.95 ± 6.35 (13.00)	<0.0001 *	Yes ^‡^

AC—arm circumference; WC—waist circumference; HC—hip circumference; WHR—waist-to-hip ratio; WHtR—waist-to-height ratio; BMI—body mass index; TBFM—total body fat mass; VFL—visceral fat level; NS—not significant; SD—standard deviation. * Kruskal–Wallis test. ⸷ ANOVA test. ^‡^ Dunn test; ^§^ Bonferroni test.

## Data Availability

The data presented in this study are available on request from the corresponding author.

## Abbreviations

AC	Arm circumference
AT	Adipose tissue
BMI	Body mass index
CVD	Cardiovascular disease
HC	Hip circumference
hsCRP	High-sensitivity C-reactive protein
IFN-γ	Interferon-γ
IL-1β	Interleukin-1β
IL-6	Interleukin-6
LGI	Chronic low-grade inflammation
NCDs	Noncommunicable diseases
NW	Normal weight
OW/OB	Overweight/obese
WBC	White blood cell count
WC	Waist circumference
WHR	Waist-to-hip ratio
WHtR	Waist-to-height ratio
SAT	Subcutaneous adipose tissue
TBFM	Total body fat mass
TNF-α	Tumor necrosis factor-α
VAT	Visceral adipose tissue
VFL	Visceral fat level
